# Fascial Nomenclature: Update 2021, Part 2

**DOI:** 10.7759/cureus.13279

**Published:** 2021-02-11

**Authors:** Bruno Bordoni, Allan R Escher, Filippo Tobbi, Bruno Ducoux, Serge Paoletti

**Affiliations:** 1 Physical Medicine and Rehabilitation, Foundation Don Carlo Gnocchi, Milan, ITA; 2 Anesthesiology/Pain Medicine, H. Lee Moffitt Cancer Center and Research Institute, Tampa, USA; 3 Osteopathy, Poliambulatorio Medico e Odontoiatrico, Varese, ITA; 4 Osteopathy, FROP Formation Recherche Osteopathie Prévention, Bordeaux, FRA; 5 Osteopathic Medicine, Académie d'Ostéopathie de France, Paris, FRA

**Keywords:** fascia, fascintegrity, osteopathic, fascial system, fascial continuum

## Abstract

The fascial continuum is a topic of debate, in particular, its classification into a nomenclature that researchers and medical figures can agree on. Most likely, the difficulty in finding the uniqueness of this topic lies in the fact that only some scientific figures with certain specialties write articles to state their point of view. We know, however, that a matter that involves the human body cannot be taken into consideration only by some scientific arguments, but by all the notions capable of completing a multidisciplinary and impartial vision. The fascia, too often, becomes a destination for earning and selling, to the detriment of the entire scientific community. The second part of the article on fascial nomenclature tries to obtain a new definition of what could be considered the fascial continuum, based on the most innovative information in the literature; the ultimate goal is to give free reflections on the subject in full intellectual freedom.

## Introduction and background

The fascial continuum is a fascinating and ever-changing subject. Despite the constant scientific information that appears in the panorama of scientific literature, there is hardly a change of direction in what is considered the fascial tissue that is in parallel with innovation. New developments do not always meet the endorsement of some organizations; nevertheless, as often happens in clinical practice, the change of opinion is not immediate: “Sometimes, long-held beliefs must be challenged and disregarded” [1]. Understanding and identifying fascial tissue is extraordinarily important for highlighting the clinical cause in various pathological aspects.

According to a recent research on the cause of occipital neuralgia, about 98% of patients undergoing greater occipital nerve decompression surgery show a non-physiological adaptation of the connective tissue of the trapezius muscle, a change that would represent one of the main causes of the presence of headache and migraine [2]. The tissue is fibrotic, not very elastic, and unable to allow the nerve to flow correctly between the different tissues. The capacity of the structure that supports and contains the peripheral nervous tissue, that is, the fascial tissue, must be able to adapt to the different mechanical stresses during the movement of the trunk and/or limbs; if this does not happen, the fascial tissue of the nerve can become a source of neuralgia [3].

A rarer pathological event is the growth of tumor masses within the connective tissue, both muscular and visceral. Ligamentoid fibromatosis is a mesenchymal neoplasm that is more easily found in women; surgical treatment is the preferred approach [4]. This tumor form can be confused with stromal cancer when it is present in the abdominal area [4]. It becomes essential to understand the qualities of the fascial tissue examined.

Connective tissue could be a cause of widespread pain in people accustomed to running before the symptom becomes evident. A recent study has highlighted changes in areas of the lower limb that have undergone a non-physiological adaptation of the runner, such as the patellar area, the plantar arch of the foot, and the tibial portion where the fascial complex of the lateral thigh musculature is attached; these changes could be one of the most important causes of the pain reported by the athlete and one of the causes of the finding altered biomechanics of the gait [5].

The pain of the plantar arch is found not only in athletes but also in non-sports people. Causes related to this fascial problem are alterations in the myofascial structure of the lower limb (thigh and/or leg) and alterations in the structure of the plantar fascia (fibrosis, thickening [6,7]. A change in the thickness and constitution of the fascial tissue will make the action of the limb more difficult, as well as the functions of an organ (abdominal or thoracic) constituted or enveloped by the fascial continuum [8,9]. We must remember that the fascial tissue is of vital importance in the transmission of mechanical information, not only to carry out a movement but also to allow all tissues to be able to handle the change imposed by internal and external physiological stressors [10,11].

Another essential role related to the fascial continuum is to correctly convey the body fluids, creating the space and pressure sufficient for the passage of fluids; fascia creates a constant connection between all areas of the body, like a fluidic network [12]. The same body fluids are part of the connective tissue or liquid fascia, which are essential to carry mechano-metabolic information in all body parts, from the largest to the smallest, up to the cell [13].

The second part of the article reviews the embryological information on fluid connective tissue, with clinical reflections to emphasize the importance of the fascial fluid aspect. The article concludes its journey with a new definition of what should be considered as fascial tissue, in the light of the most recent scientific notions and with a multidisciplinary perspective. The updated fascial nomenclature derives from the conclusions of various health and research figures from the academic and scientific world under the aegis of the Foundation of Osteopathic Research and Clinical Endorsement (FORCE) organization.

## Review

Fascial tissue embryology: liquid fascia

Who decides which type of fascial tissue to consider and which not to consider? Probably, depending on the profession or specialty, one aspect of the fascial tissue will be taken into account instead of others. Osteopathy, which has a predominant aspect in FORCE, has a multidisciplinary approach and with manual techniques capable of acting on all fascial aspects [14,15]. The blood, lymph, and cerebrospinal fluid (CSF) represent the liquid fascia. These body fluids derive from the mesoderm and to a small extent from the ectoderm [15,16]. In particular, the CSF initially derives from the placental fluid, and as the development and formation of the central nervous system continues, the CSF will be produced by the mesoderm [16]. When the embryonic period ends with the presence of sketches of the choroid plexuses (41 days of gestation), the CSF will be synthesized from areas connected to the ectoderm (neuroepithelium) [16]. About 99% of CSF is water and only about 1% is body fluid [16]. When produced by the neuroepithelium, CSF exerts expansive forces to stimulate the same neuroepithelium to grow; moreover, the brain cavities that are forming have an extracellular matrix rich in chondroitin sulfate proteoglycan, which, under growth stimulation, retain water, influencing hydrostatic pressures and further brain growth [17].

Hemangiogenic progenitor cells deriving from mesoderm, thanks to the presence of fetal liver kinase 1 (Flk1), are able to stimulate blood formation, the endothelium of vessels, and smooth muscle cells and cardiomyocytes [18]. The corpuscular portion of fluids, such as blood, is able to change its volume (by trafficking intracellular ions), so as to adapt to the volume of the spaces in which they are present and travel [19]. Body fluids or liquid fascia are responsible for the morphogenic and morphogenetic stimulus [20]. To give some examples, the movement of fluids in the bone canaliculi in the presence of mechanical load stimulates the adaptation processes and the osteocytes to maintain the growth/decrease balance between osteoblasts and osteoclasts [21]. The blood flow through mechanical stimulation towards the cells (shear stress), is able to stimulate the receptors that respond to the change in fluid pressure, improving the formation of the uterus during development [22]. Receptors essential for the release of calcium into the cell (troponin transient receptor potential type 1 and 2), triggering muscle contraction with the actomyosin complex, are stimulated thanks to the presence of shear stress on the part of the body fluids [23].

Body fluids are viscoelastic and, depending on their viscosity, are able to influence mechanical and electromagnetic oscillations; the frequency of the oscillation will depend on the degree of elasticity of the fluids, according to the physics of fluids [24,25]. Fluids determine what the mechanometabolic environment of solid tissues will be like; if the body fluids do not have correct mechanical information, the final result of the development of the tissues will be altered, with evidence of pathologies [20,26]. The lymphatic system would arise from the venous system (mesoderm) during ontogenetic processes; in mice, the lymphatic system arises from the cardinal vein, thanks to some endothelial cells that begin to form the prosperous homeobox transcription protein 1, which is a protein involved in the development of lymphatic tissue (and other structures) [27]. After the appearance of the growth factor, the presence of vascular endothelial growth factor C allows the growth and branching of the ex-endothelial cells to form the first lymphatic plexus; other non-specialized and hemogenic endothelial cells will help the formation of lymphatic tissue during embryonic lymphangiogenesis, as well as other target organ cells (heart, epidermis, intestine) [27]. During embryogenesis, body fluids, including lymph and cerebrospinal fluid, allow the stimulation of specific genes and the creation of the final body structures in the forms we know [28].

**Figure 1:**
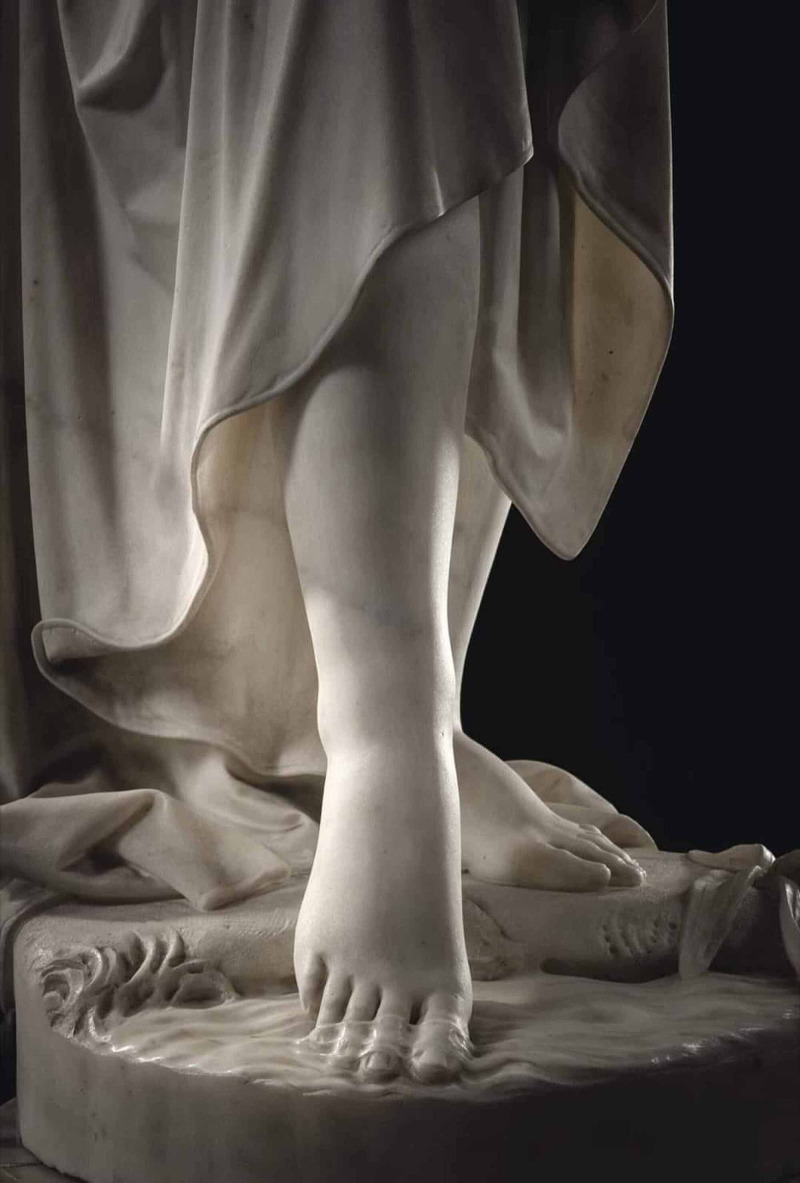
Image of Nymph statue (detail), Giovanni Battista Lombardi, 1823 -1880. Palazzo Faschi, Brescia. The detail ideally highlights the solid and liquid fascia. Photograph by Bruno Bordoni.

The fascial continuum, solid and fluid, is a web, and any stimulus present cannot escape because, regardless of the nature of the stressor, the whole network becomes aware of it and adapts. The cellular and extracellular environment are influenced by mechanical-metabolic stimuli (mechanoreciprocity), and among these stressors, we find the forces of body fluids [29]. We know that an increase in stiffness of the mammary gland tissue or an increase in collagen alters the mechano-metabolic environment, favoring the formation of tumors [29]. The increase in fluid pressure inside, around, and between cells is another important factor in the increase in the stiffness of the mammary gland (and other tissues) and is considered another potentially pathogenic factor (Figure 1) [29,30].

Fascial nomenclature 2021

There are many concepts related to the fascial continuum, such as biotensegrity and fascial chains. The first term derives from architecture or tensegrity, which word comes from the ideas of the designer R. Buckminster Fuller in 1960 [31]. The architectural model was translated into human biology, first in 1977 with the vertebral column and then with the cell structure in 1993, by Dr Robbie and Dr Ingber, respectively [31]. The word biotensegrity was coined by Dr Levin, through an abstract in 1981 at the 34th Annual Conference on Engineering in Medicine and Biology [31]. What the term biotensegrity implies is that the solid structure (bones and myofascia) are interdependent. Biotensegrity does not consider the presence of body fluids and, moreover, it is shown that a mechanical stressor does not affect the whole cell, but only in some areas. The cell membrane transports the sensed mechano-metabolic tension in a non-homogeneous way, and not all the membrane responds in unison to the same tension; a membrane deformation can activate biochemical responses only locally, or, activate distant responses, but without necessarily activating mechanotransduction where the mechanical deformation has occurred [32]. This means that the mechanotransduction phenomenon will involve the receptors or ion channels positioned in a certain area of the cell, regardless of the stimulus that deforms the membrane [32]. Here the biotensegrity ends. The term that FORCE has devised to better understand the response mechanisms of cells and tissues, involving body fluids (without which the shape and function we know would not exist) is fascintegrity [31,33,34]. The vision of a myofascial continuum, that is, a continuity of the myofascial tissue where the tension of a district influences all the "links" of the chain can be found in several authors: Busquet (1992); Souchard (1993); Myers (1997); Paoletti (1998) [35,36]. Recent studies support this view, using the schemes devised by the authors cited previously, to solve problems related to pain in the lumbar area due to vertebral degeneration and to improve athletic performance, even if we need further confirmation [37,38].

**Figure 2:**
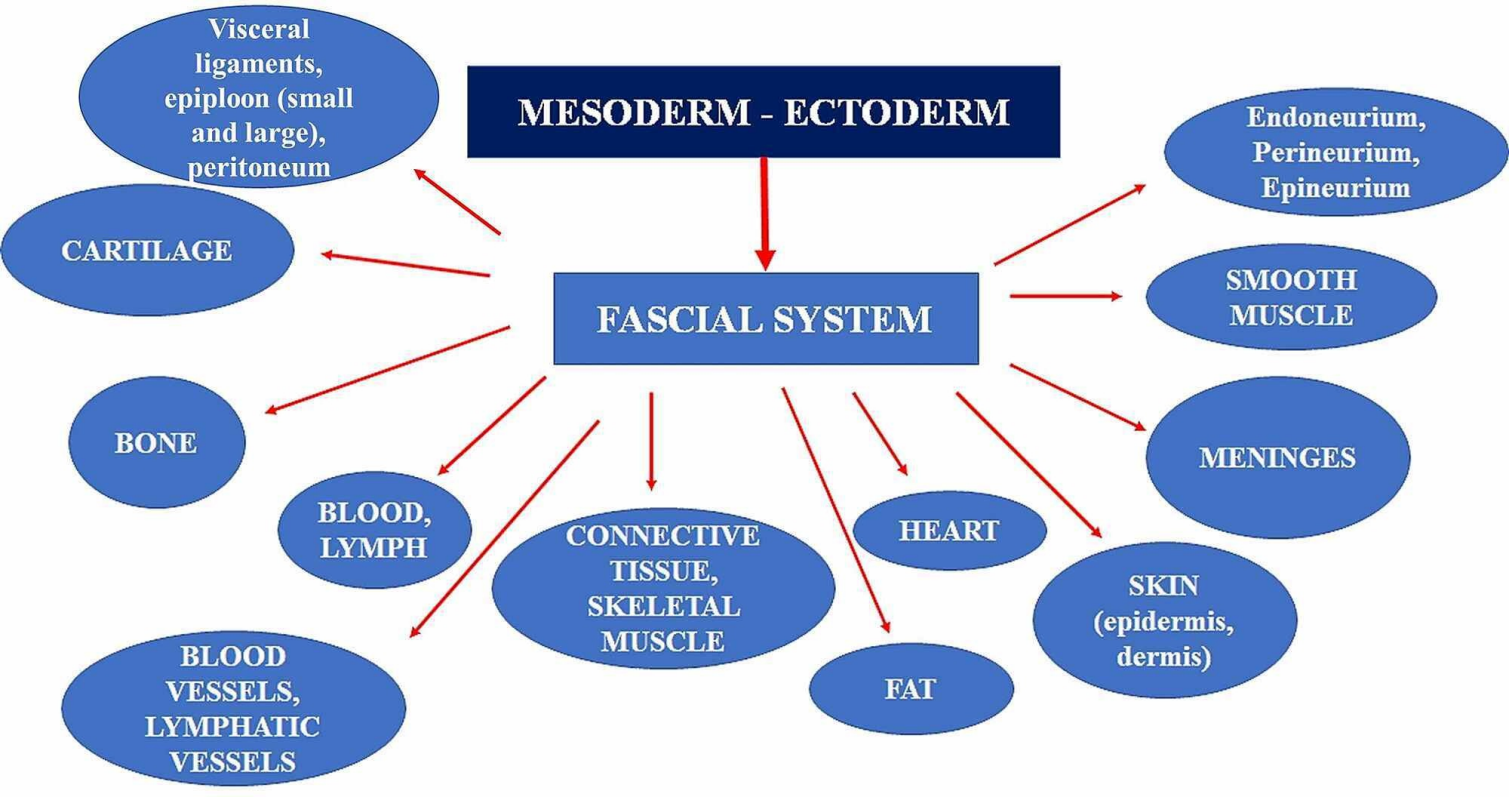
The figure illustrates the tissues that could be considered as fascial tissue, originating from the mesodermal and ectodermal sheets.

Studies are still lacking to demonstrate whether the myovisceral chains, that is the mesodermal system that connects the viscera and muscles (diaphragm, Glisson's capsule, endothoracic fascia, and other connections), have an influence on movement and on localized and distal districts [39,40]. In light of the foregoing and of the material in the literature, according to the vision of FORCE and from what has been published previously, we describe what could be considered as an updated fascial nomenclature: the fascia is any tissue that contains features capable of responding to mechanical stimuli. The fascial continuum is the result of the evolution of the perfect synergy among different tissues, liquids, and solids, capable of supporting, dividing, penetrating, feeding, and connecting all the districts of the body: epidermis, dermis, fat, blood, lymph, blood and lymphatic vessels, tissue covering the nervous filaments (endoneurium, perineurium, epineurium), voluntary striated muscle fibers and the tissue covering and permeating it (epimysium, perimysium, endomysium), ligaments, tendons, aponeurosis, cartilage, bones, meninges, involuntary striated musculature and involuntary smooth muscle (all viscera derived from the mesoderm), visceral ligaments, epiploon (small and large), peritoneum, and tongue. The continuum constantly transmits and receives mechano-metabolic information that can influence the shape and function of the entire body. These afferent/efferent impulses come from the fascia and the tissues that are not considered as part of the fascia in a bi-univocal mode (Figure 2) [41].

Further studies will have to be carried out to fully understand the functions and descriptive aspects of the fascial continuum; certainly, the research must always be free from economic interests, or the result will always be biased: “Research should always be free from lucrative financial intentions, just as researchers should not have the objective of earning money by limiting the knowledge and clinical application of information to the exclusion of other health professionals” [42]. Knowing the fascia allows us to better understand the patient's need, since not only the fascial continuum influences the solid and liquid portion of the body, but also the emotional sphere and the subjective perception of pain [43].

## Conclusions

Compared to the previous scientific publications of the FORCE group, which includes scholars and professionals from all over the world and with different academic qualifications, we have added anatomical structures not mentioned so far, such as visceral ligaments, epiploon (small and large), and peritoneum. Further research and articles will be needed to understand and classify the different components of the fascial continuum. No science can be defined as having reached the conclusion of its understanding. We conclude with a final reflection, that is, knowledge is not a point of arrival but, rather, a broad basis for finding further questions.
